# Delaying age at first sexual intercourse provides protection against oral cavity cancer: a mendelian randomization study

**DOI:** 10.3389/fonc.2024.1361527

**Published:** 2024-04-18

**Authors:** Ting Sun, Xin He, Xing Chen, Yang Huaqing, Haimei Zhang, Min Zhao, Li Du, Bin Zhao, Junping Hou, Xudong Li, Yu Liu

**Affiliations:** ^1^ Department of Oncology, Guangyuan Central Hospital, Guangyuan, China; ^2^ Department of Emergency, Guangyuan Central Hospital, Guangyuan, China; ^3^ Department of Dentistry and Oral Surgery, Guangyuan Central Hospital, Guangyuan, China; ^4^ Department of Dentistry and Oral Surgery, North Sichuan Medical College, Nanchong, China

**Keywords:** oral cavity cancer, Mendelian randomization, sexual intercourse, epidemiology, malignant tumor

## Abstract

**Aim:**

To investigate whether age at first sexual intercourse could lead to any changes in the risk of oral cavity cancer.

**Methods:**

A two-sample mendelian randomization was conducted using genetic variants associated with age at first sexual intercourse in UK biobank as instrumental variables. Summary data of Northern American from a previous genome-wide association study aimed at oral cavity cancer was served as outcome. Three analytical methods: inverse variance-weighted, mendelian randomization Egger, and weighted median were used to perform the analysis, among which inverse variance-weighted was set as the primary method. Robustness of the results was assessed through Cochran Q test, mendelian randomization Egger intercept tests, MR PRESSO, leave one out analysis and funnel plot.

**Results:**

The primary analysis provided substantial evidence of a positive causal relationship age at first sexual intercourse and the risk of oral cavity cancer (p = 0.0002), while a delayed age at first sexual intercourse would lead to a decreased risk of suffering oral cavity cancer (β = -1.013). The secondary outcomes confirmed the results (all β < 0) and all assessments supported the robustness, too (all *p* > 0.05).

**Conclusion:**

The study demonstrates that a delayed sexual debut would provide protection against OCC, thus education on delaying sexual intercourse should be recommended.

## Introduction

Oral cavity cancer (OCC), a prevalent form of head and neck cancer, encompasses neoplasms originating in various areas, including the lips, the anterior two-thirds of the tongue, gingivae, hard and soft palate, oral mucosal surfaces, and floor of the mouth (1, 2). Oral squamous cell carcinoma accounts for more than 95% of OCC cases, constituting 2% of all malignant lesions and resulting in over 30000 new cases annually (3). The incidence of OCC is higher in men than in women, and it is typically diagnosed around the age of 60. However, recent trends indicate a rising incidence among women and young adults, attributed to specific risk factors (1, 4, 5). Despite the highest prevalence in Asia, Europe, notably Hungary, experiences persistently high age-standardized incidence and death rates for OCC, with no significant decline observed in recent decades (6).

The 5-year survival rate for oral cancer remains low, even with advancements in both diagnosis and treatment (7). Therefore, the emphasis on early control and intervention of pathogenic factors is paramount. Substantial evidence supports smoking, alcohol consumption, and Human Papillomavirus (HPV) infection as confirmed risk factors for OCC. Additionally, a family history of OCC, lower body mass index, an inadequate diet with insufficient fruit and vegetable intake, type 2 diabetes, poor oral health, lower educational attainment, and occupational factors are regarded as less established risk factors (8).

Interestingly, some studies have linked sexual behavior, including age at first intercourse, to the development of OCC (9, 10). In a case report, OCC in a young patient was suspected to be related to premature marriage and early sexual intercourse (11). A previous case control study pointed out that oral squamous cell carcinoma risk increased with self-reported decreasing age at first intercourse (12). However, this conclusion was not supported by following similar studies (13, 14). Even systematic review admitted that there were too few studies to draw conclusions on this topic (10). Therefore, whether early sexual intercourse will cause an increase in the prevalence of OCC remains controversial.

In addition to the inconsistency in conclusions, existing studies were mainly case reports or case control studies, which did not have the ability to uncover causal relationships. Given the impracticality of designing a randomized controlled trial for this issue, the role of mendelian randomization (MR) should increasingly come to the forefront in this type of research.

Mendelian randomization studies use genetic variants robustly related to exposures as instrumental variables (IV) to detect the influence of the exposure on various outcomes. It has a unique advantage in revealing causal relationships since the allocation of genetic variants is almost random, thus reducing potential bias from confounding and reverse causation (15, 16). MR relies on 3 assumptions, relevance, independence and exclusion restriction assumptions (17). Fulfillment of these assumptions can ensure that the impact of IVs on outcomes comes only from exposure factors. Because of the above advantages, MR is widely used in the field of epidemiology.

Based on the above background, this study aims to investigate whether age at first intercourse could lead to any changes in the risk of OCC through MR.

## Methods

This is a two sample MR study using data collected from open-source database. Owing to the use of open data, this study did not require ethical approval and the informed consent were provided in the original publications and these publicly available databases. This study has not been registered anywhere. The work was reported following the strengthening the reporting of observational studies in epidemiology using mendelian randomization (STROBE-MR). The codes for analysis are available from the authors upon request.

Data of exposure came from UK biobank. The genome-wide association study (GWAS) identified single-nucleotide polymorphisms (SNP) associated with age at first sexual intercourse in a cohort of 406457 people, predominantly European. In this study, age at first intercourse was considered a continuous variable. As for outcome, another GWAS shared their data of OCC in 6034 cases and 6585 controls, of which more than 90% were predominantly of European ancestry (18). To ensure homogeneity and nonoverlap of two samples, only data of participants with OCC alone in North America was extracted from this study. Finally, the datasets of outcome comprised 1135 cases and 2329 controls. It is worth mentioning that, in order to ensure the exclusion restriction assumption, this study also used GWAS data of some certain risk factors of OCC, including smoking, alcohol and HPV infection (19, 20). Similar to the study above, Europeans dominated samples of these GWAS.

All datasets were accessed online through IEU open GWAS project, and the GWAS ID of age at first sexual intercourse, OCC, smoking, alcohol and 2 high-risk human HPV infections were ukb-b-6591, ebi-a-GCST012238, ieu-b-4877, ukb-a-226, prot-c-2623_54_4 and prot-c-2624_31_2, respectively on the website.

The IVs were SNPs extracted from the GWAS summary of the exposure set with p < 5×10^−8^, ensuring compliance with the relevance assumptions. Subsequently, these SNPs underwent a check for linkage disequilibrium, with an r^2^ threshold set at 0.001. Following the removal of SNPs exhibiting linkage disequilibrium, the GWAS summary of the outcome was searched for the remaining SNPs, and their relevant information was extracted. It is noteworthy that SNPs not included in the GWAS of the outcome or those with a p < 5×10^−6^ were discarded. Any SNPs significantly associated (p < 5×10^−8^) with specific risk factors of OCC were also extracted from related GWAS summaries and excluded from the outcome set to meet the exclusion restriction assumption. The remaining SNPs were adjusted to align in the same direction in both the exposure and outcome sets, while those with ambiguous directions due to palindromic sequences were removed. Finally, these SNPs were utilized in the MR analysis, employing three analytical methods: inverse variance-weighted (IVW), MR-Egger, and weighted median. Random effects IVW was designated as the primary method. A two-sided p-value < 0.05 was considered statistically significant.

Although the three basic assumptions were guaranteed during the analysis stage, it was still necessary to assess the robustness of the results, especially if there was horizontal pleiotropy. Thus, Cochran Q test, MR-Egger intercept tests, MR PRESSO, leave one out analysis and funnel plot were performed to evaluate the reliability of the main results (21, 22).

All the mentioned analyses were performed using a TwoSampleMR package in R software vision 4.3.0. and R studio software.

## Results

There were 200 SNPs that met the relevance assumption in the initial searching, of which 192 could also be extracted from outcome set. However, one of these potential IVs, rs993700, was significantly associated with smoking. In order to ensure the exclusion restriction assumption and avoid horizontal pleiotropy, this SNP was removed. The rest SNPs were used as IVs to perform the following MR analysis. Since most participants were Europeans and came from different countries, as described above, the similarity of the genetic variant-exposure associations between the exposure and outcome samples were ensured, as well as the sample overlap was avoided. Thus, these IVs can be a good representation of the exposure sample and used in the analysis of outcome set. The information of selected IVs was listed in **Supplementary Material 1**.

**Figure 1:**
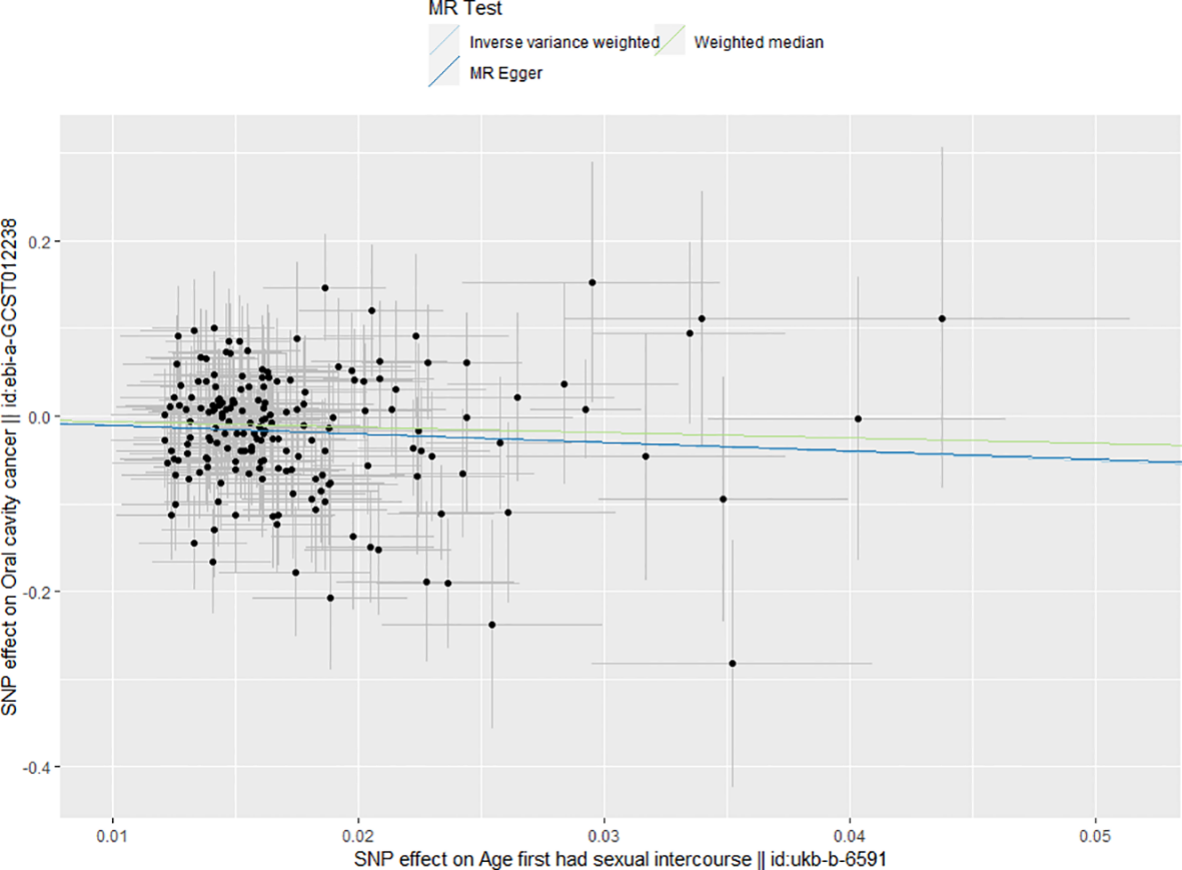
Main results of mendelian randomization. The results of all three MR analytical methods indicated every increase in age at first sexual inter course could decrease the risk of OCC.

The primary results showed that age at first sexual intercourse was significantly associated with the risk of OCC (*p* = 0.0002), and a delayed age at first sexual intercourse would lead to a decreased risk to suffer OCC (β = -1.013). This result was also confirmed by secondary results (all β < 0), indicating the reliability of the analysis. The results were shown in **Table 1** and visualized in **Figure 1**.

**Table 1:** The main results of mendelian randomization.

Method	NSNP	β	SE	*p*
Inverse Variance Weighted	181	-1.013	0.275	0.000
Weighted Median	181	-0.611	0.411	0.137
Mendelian Randomization Egger	181	-0.950	1.270	0.456

NSNP, number of single-nucleotide polymorphisms; SE, standard error.

The main results passed the heterogeneity test with *p* = 0.454 and 0.433 in the Cochran’s Q test for IVW and MR Egger analyses, respectively, which indicated the homogeneity among different IVs. The results were shown in **Table 2**.

**Table 2:** The results of Cochran’s Q test.

Method	Q	Q degree of freedom	*p*
Mendelian Randomization Egger	181.522	179	0.433
Inverse Variance Weighted	181.524	180	0.454

**Figure 2:**
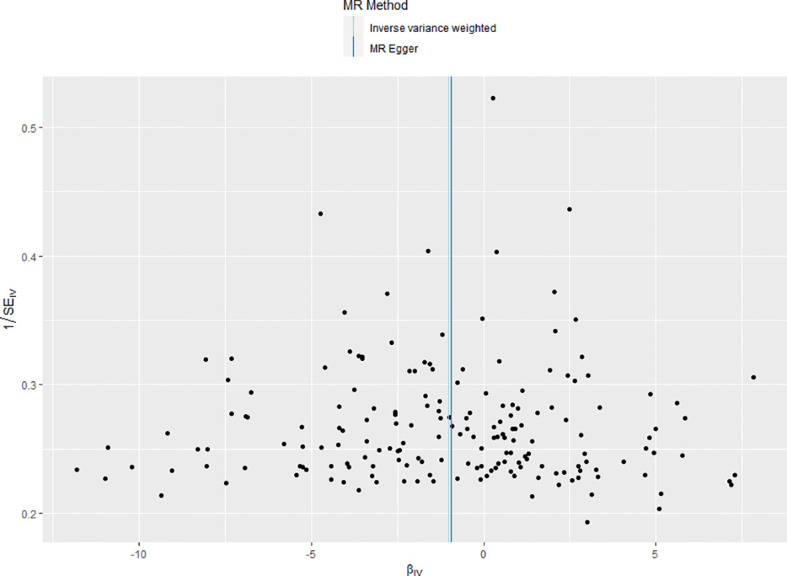
Funnel plot of the included instrumental variants. The instrumental variants evenly distributed around the mean line, indicating no bias of the instrumental variants.

Furthermore, the funnel plot provided additional confirmation of homogeneity among various IVs, as they were evenly distributed around the mean. The results were shown in **Figure 2**.

Moreover, the MR-Egger intercept test did not reveal any evidence of horizontal pleiotropy (*p* = 0.959), either. When the effect of SNPs on exposure did not exist, the estimate of their effects on the outcome is -0.001, indicating there is little horizontal pleiotropy at all. The results were shown in **Table 3**.

**Table 3:** The results of MR-Egger intercept test.

Egger_Intercept	Standard Error	*p*
-0.001	0.021	0.959

**Figure 3:**
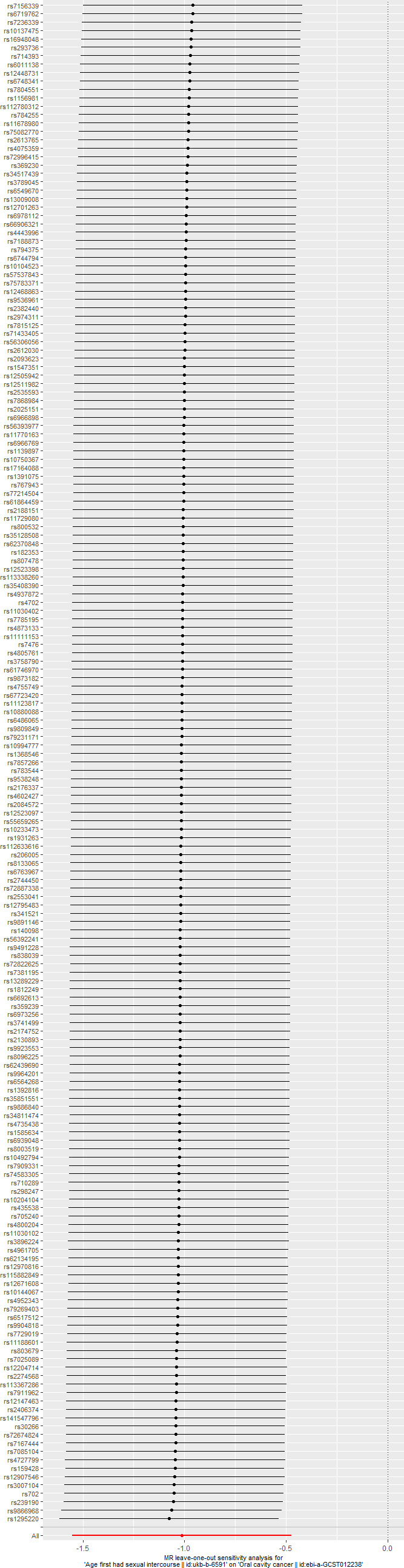
The results of leave one out test. removal of any single-nucleotide polymorphism will not change the initial results.

Additionally, the leave one out test indicated that the removal of any SNP could not significantly impact the results. This indicated that the result was not caused by a certain SNP and was robust. The results were shown in **Figure 3**.

Finally, the MR presso test produced a result of *p* = 0.465, suggesting the absence of outliers among the selected IVs.

## Discussion

The present study demonstrated that an earlier age at first sexual intercourse would lead to an increased risk of OCC. The trend in the results did not change with the analytical methods changing. In addition, none of the evaluations of the robustness of the results yielded positive results. Thus, the results were so reliable.

The relationship between age at first sexual intercourse and incidence of OCC has attracted attention many years ago. In 1998, a case-control study reported a significant increase in risk of OCC among men if they have regular intercourse before 18 years old, while the significance did not exist in women (12). Another following case-control study on oropharyngeal cancer found that an age of 17 years or younger at first sexual intercourse was associated with oropharyngeal cancer in HPV 16 positive patients, but not in the whole population (13). The combined effect of HPV 16/18 and age at first intercourse on oral squamous cell carcinoma was also observed in Chinese population (14). However, in addition to insufficient literature, the evidence on relationship between age at first sexual intercourse and OCC in whole population were still inconsistent, while fewer documents supported the establishment of this correlation. The present study added to the existing evidence and confirmed that existence of correlation between age at first sexual intercourse and OCC. Moreover, the majority of the existing research consisted of case-control studies, which lacked the ability to reveal causal effects. Compared to them, the present study could not only verify the correlation, but also reveal the causality between behavior and disease. Thus, early prevention became possible. Furthermore, this study had the highest level of evidence among existing studies, as MR studies were second only to systematic reviews and randomized controlled trials in terms of level of evidence (23). In view of the difficulty in implementing RCT on this topic, the results of this study might also be the most solid evidence on the causality between age at first sexual intercourse and OCC in the future.

The most well-established risk factors of OCC include smoking, alcohol consumption, and HPV infection (8). In order to eliminate the impact of the horizontal pleiotropy of IVs on these factors on the research results, all IVs that are strongly correlated with them were excluded. This ensured that these factors did not directly affect the experimental results beyond the exposures we studied, while this does not prevent them from acting as mediators between exposures and outcomes. It is not difficult to guess that the most relevant to sexual behavior among these factors, is HPV infection, which may be caused by sexual intercourse and leads to OCC, and the idea that oral HPV infection could come from sexual behavior has also been confirmed previously (13).

HPV is one of the most prominent infectious agents causing cancer (24). It belongs to the family *Papillomaviridae* and consists of non-enveloped virons containing a double-stranded DNA genome. Enclosed within an icosahedral capsid, this genetic material is composed of major and minor structural proteins, namely L1 and L2, respectively. Demonstrating high tissue specificity, these viruses infect both cutaneous and mucosal epithelium. Utilizing the genomic sequence of L1, the gene responsible for the principal capsid protein, researchers have identified and characterized over 200 HPV types. Among them, at least 14 are classified as high-risk types that may lead to cancer, especially types 16 and 18 (25, 26). Cervical cancer is most susceptible to HPV and nearly all cervical cancer cases are related to HPV infection (26). The association of high-risk HPV and OCC was also observed in many studies among different population and widely recognized (14, 27, 28).

The specific transmission routes of HPV are also of great significance to the prevention and control of OCC. Although it can be transmitted through contact, sexual transmission, obviously including oral sex, is the main route of HPV infection (29–31). In terms of oral cavity, HPV colonizes the oral cavity in various ways. Among them, there are two main ways for HPV infected through sexual intercourse to colonize the oral cavity. Autoinoculation between genital, oral, or anal sites may occur through intermediate contact with hands or virus shedding in the anogenital region (32). Women with proven cervical HPV infection have a higher prevalence of HPV in oral samples, indicating there might be a relationship between such infections (33). In addition to autoinoculation, engaging in oral sex and open-mouthed kissing, behaviors that have become increasingly common, are also associated with the development of oral HPV infection (34, 35).

Another factor that contributes to the protective effect of delayed sexual intercourse against OCC may be vaccination. Vaccination against HPV substantially reduces the risk of HPV associated cancers, including OCC (36). Apparently, people who have sex for the first time after being vaccinated will benefit from this protective factor, and this results in differences in the chances of contracting HPV between people who lose their virginity early and those who lose their virginity later. Given that timing of vaccination is a hidden modifiable variable, this finding may help reduce the incidence of OCC.

Some limitations of the present study should also be mentioned. First, the work only demonstrated that a delay sexual debut might lead to a decrease in the risk of OCC but did not detect the underlying mechanism. Based on previous literature, HPV and vaccination might play an important role in this causal chain, thus more research in this area is necessary in the future. Second, since both samples in this study were strictly restricted to European populations, whether this conclusion is valid for other populations remains to be discussed, though observational studies indicated the effect might work in other populations as well (14). Considering that the susceptibility and manifestations of OCC vary among different populations, and culture of different areas also influences the incidence of oral sex, similar validation studies for other populations or other regions are also necessary (37, 38).

Despite limitations, the work has clinical relevance and contributes to public policy. The preventive measures of OCC can be classified as primary, secondary and tertiary preventions, while the primary prevention is to improve public awareness of risk factors, thus to avoid habits that may lead to cancer (39). Since this study reveals the causal relationship between age at first sexual intercourse and OCC, and age at first sexual intercourse is a modifiable variable, OCC could be protected by educating adolescents to delay sexual debut, in addition to staying away from tobacco and alcohol. In addition to age at first sexual intercourse, the vaccine is another modifiable factor that can block the causal connection of occurrence of the two traits. Based on the existing knowledge, future studies should focus on 3 issues. First, the results in this study based on the European population should be verified in other populations before they can be generalized, while this also calls for more GWAS. Second, sociological research on intervention strategies to delay sexual debut or to make vaccination time earlier as preventive measures to OCC would be significant. Third, basic researcher on the underlying mechanism by which HPV causes OCC is still needed.

## Conclusions

This study demonstrates that a delayed sexual debut would provide protection against OCC. Thus, education on delaying sexual intercourse and early vaccination against HPV should be recommended in terms of preventing of OCC. However, considering the intricate interplay of sexual behavior, consent, and health, further exploration of how this recommendation aligns with broader sexual health education programs, would be valuable.

## Data availability statement

The original contributions presented in the study are included in the article/**Supplementary Material**. Further inquiries can be directed to the corresponding author.

## Author contributions

TS: Conceptualization, Methodology, Supervision, Writing – original draft. XH: Conceptualization, Formal analysis, Methodology, Writing – original draft. XC: Conceptualization, Writing – original draft. YH: Formal analysis, Software, Writing – original draft. HZ: Formal analysis, Software, Writing – original draft. MZ: Visualization, Writing – original draft. LD: Validation, Writing – original draft. BZ: Visualization, Writing – original draft. JH: Writing – original draft. XL: Investigation, Writing – original draft. YL: Project administration, Writing – original draft.
