# Supplementary material for: Delaying age at first sexual intercourse provides protection against oral cavity cancer: a mendelian randomization study
Source: Front Oncol. 2024 Apr 18;14:1361527. doi: 10.3389/fonc.2024.1361527 (PMC11063229; doi:10.3389/fonc.2024.1361527)
Supplement: Supplementary file 1 [file Table_1.docx]

Supplemental material table 1. Information of selected SNPs

| SNP | effect allele | other allele | beta | se | effect allele frequency | chr | pos | p |
| --- | --- | --- | --- | --- | --- | --- | --- | --- |
| rs10104523 | C | T | -0.013 | 0.002 | 0.522 | 8 | 73889570 | 6.29999e-09 |
| rs10137475 | A | G | 0.013 | 0.002 | 0.577 | 14 | 58797953 | 1.09999e-09 |
| rs10144067 | T | C | -0.014 | 0.002 | 0.591 | 14 | 93885198 | 1.5e-10 |
| rs10204104 | A | C | -0.016 | 0.003 | 0.832 | 2 | 1.74E+08 | 1.5e-08 |
| rs10233473 | T | C | -0.014 | 0.002 | 0.252 | 7 | 32265007 | 7.49998e-09 |
| rs10492794 | T | C | 0.016 | 0.003 | 0.178 | 16 | 12639850 | 1.2e-08 |
| rs10496949 | C | G | 0.015 | 0.002 | 0.578 | 2 | 1.44E+08 | 8.19974e-12 |
| rs10750367 | G | A | -0.015 | 0.002 | 0.554 | 11 | 1.27E+08 | 3.59998e-12 |
| rs10880088 | T | C | 0.016 | 0.003 | 0.216 | 12 | 41905897 | 8e-10 |
| rs10922907 | T | A | 0.022 | 0.002 | 0.549 | 1 | 91193049 | 4.10015e-25 |
| rs10994777 | A | G | 0.019 | 0.003 | 0.162 | 10 | 63233988 | 1.40001e-10 |
| rs11030102 | G | C | -0.014 | 0.002 | 0.261 | 11 | 27681596 | 8.19993e-09 |
| rs11030402 | G | A | 0.013 | 0.002 | 0.368 | 11 | 28676170 | 4.49997e-09 |
| rs11111153 | C | A | -0.014 | 0.003 | 0.245 | 12 | 1.02E+08 | 2.59998e-08 |
| rs11123817 | T | C | 0.015 | 0.002 | 0.401 | 2 | 1.01E+08 | 6.59933e-12 |
| rs11188601 | C | T | -0.017 | 0.002 | 0.365 | 10 | 97877900 | 1.59993e-14 |
| rs112633616 | C | A | 0.04 | 0.006 | 0.033 | 12 | 23238326 | 3.10027e-11 |
| rs112780312 | A | G | -0.017 | 0.002 | 0.275 | 1 | 1.54E+08 | 1.29987e-11 |
| rs113338260 | C | T | -0.016 | 0.003 | 0.216 | 5 | 46004640 | 2.5e-09 |
| rs113367286 | T | C | 0.02 | 0.002 | 0.278 | 7 | 1.4E+08 | 1.99986e-16 |
| rs1139897 | A | G | 0.018 | 0.003 | 0.224 | 16 | 720986 | 1.20005e-11 |
| rs1156981 | G | A | -0.023 | 0.004 | 0.902 | 1 | 88829969 | 2.90001e-10 |
| rs115882849 | A | G | -0.019 | 0.003 | 0.153 | 3 | 54156724 | 1.7e-10 |
| rs11678980 | A | G | -0.012 | 0.002 | 0.461 | 2 | 1.62E+08 | 2.90001e-08 |
| rs11729080 | A | G | 0.023 | 0.003 | 0.171 | 4 | 1.13E+08 | 9.20026e-16 |
| rs11770163 | C | G | -0.013 | 0.002 | 0.332 | 7 | 1.16E+08 | 1.40001e-08 |
| rs12147463 | A | G | -0.021 | 0.003 | 0.193 | 14 | 41059928 | 3.90032e-14 |
| rs12204714 | T | C | 0.029 | 0.002 | 0.632 | 6 | 1.52E+08 | 4.30031e-39 |
| rs1226414 | T | A | 0.016 | 0.002 | 0.505 | 2 | 1.57E+08 | 6.00067e-13 |
| rs12448731 | T | C | -0.017 | 0.003 | 0.142 | 16 | 49622284 | 1.89998e-08 |
| rs12468863 | T | C | 0.014 | 0.002 | 0.518 | 2 | 26940294 | 4.09996e-10 |
| rs12505942 | C | T | 0.016 | 0.002 | 0.343 | 4 | 1.41E+08 | 1.69981e-12 |
| rs12511982 | A | G | 0.013 | 0.002 | 0.551 | 4 | 60736871 | 5.89997e-09 |
| rs12523097 | C | T | -0.016 | 0.002 | 0.298 | 5 | 1.67E+08 | 1.10002e-11 |
| rs12523398 | A | T | 0.023 | 0.003 | 0.174 | 5 | 45119647 | 2.99985e-15 |
| rs12653396 | A | T | -0.022 | 0.002 | 0.565 | 5 | 87847273 | 1.29987e-23 |
| rs12671608 | C | T | -0.018 | 0.003 | 0.139 | 7 | 1.54E+08 | 2.19999e-08 |
| rs12701263 | T | C | 0.014 | 0.002 | 0.4 | 7 | 32962089 | 6.09958e-11 |
| rs12795483 | G | A | 0.013 | 0.002 | 0.518 | 11 | 79883503 | 2.19999e-09 |
| rs12907546 | A | G | -0.023 | 0.003 | 0.212 | 15 | 47684280 | 7.39946e-18 |
| rs1295220 | T | C | -0.019 | 0.002 | 0.253 | 5 | 1.55E+08 | 6.70039e-14 |
| rs12970816 | A | G | -0.015 | 0.002 | 0.395 | 18 | 50023859 | 5.50047e-12 |
| rs13009008 | G | A | 0.018 | 0.002 | 0.672 | 2 | 1.74E+08 | 1.59993e-15 |
| rs13289229 | T | G | 0.018 | 0.003 | 0.178 | 9 | 86224419 | 4.49997e-10 |
| rs1368546 | C | T | 0.016 | 0.002 | 0.557 | 2 | 1.04E+08 | 1.59993e-13 |
| rs1391075 | C | A | -0.015 | 0.002 | 0.643 | 12 | 84052203 | 8.49963e-12 |
| rs1392816 | T | C | 0.018 | 0.002 | 0.388 | 1 | 66481188 | 3.80014e-15 |
| rs140098 | C | T | -0.012 | 0.002 | 0.548 | 22 | 30126644 | 1.5e-08 |
| rs141547796 | A | G | 0.033 | 0.004 | 0.082 | 6 | 50615935 | 2.80027e-17 |
| rs1454687 | G | C | 0.012 | 0.002 | 0.515 | 3 | 94038085 | 2.80001e-08 |
| rs1547351 | A | T | 0.012 | 0.002 | 0.41 | 8 | 36842153 | 2.5e-08 |
| rs1585634 | C | G | -0.016 | 0.003 | 0.802 | 8 | 54396376 | 1e-08 |
| rs159428 | C | T | -0.013 | 0.002 | 0.526 | 20 | 31099311 | 7.19996e-10 |
| rs16948048 | G | A | -0.017 | 0.002 | 0.368 | 17 | 47440466 | 8.99912e-14 |
| rs17164088 | G | A | -0.012 | 0.002 | 0.422 | 4 | 2675893 | 1.40001e-08 |
| rs1812249 | A | G | -0.015 | 0.003 | 0.215 | 1 | 1.12E+08 | 1.40001e-08 |
| rs182353 | T | C | -0.013 | 0.002 | 0.468 | 8 | 1.15E+08 | 1.7e-09 |
| rs1891588 | C | G | -0.012 | 0.002 | 0.553 | 1 | 1.52E+08 | 3.29997e-08 |
| rs1931263 | T | G | -0.012 | 0.002 | 0.49 | 1 | 96177073 | 1.79999e-08 |
| rs2025151 | G | C | 0.015 | 0.003 | 0.196 | 9 | 99161512 | 3.29997e-08 |
| rs206005 | T | A | -0.014 | 0.003 | 0.76 | 6 | 1.64E+08 | 1.2e-08 |
| rs2084572 | G | A | 0.015 | 0.002 | 0.45 | 3 | 17315758 | 1e-11 |
| rs2093623 | A | G | 0.015 | 0.002 | 0.496 | 10 | 10922977 | 5.50047e-12 |
| rs2130893 | C | G | 0.015 | 0.002 | 0.285 | 10 | 1.34E+08 | 1.5e-09 |
| rs2174752 | T | G | -0.014 | 0.002 | 0.451 | 13 | 69332015 | 3.90032e-11 |
| rs2176337 | T | A | -0.018 | 0.002 | 0.315 | 9 | 1.09E+08 | 1.9002e-14 |
| rs2188151 | T | G | -0.022 | 0.002 | 0.425 | 3 | 50201924 | 2.49977e-24 |
| rs2274568 | A | G | -0.014 | 0.002 | 0.581 | 1 | 1.11E+08 | 2.99999e-10 |
| rs2382440 | C | G | -0.017 | 0.003 | 0.156 | 9 | 14140575 | 6.29999e-09 |
| rs239190 | T | C | 0.015 | 0.002 | 0.532 | 6 | 1.01E+08 | 2.60016e-12 |
| rs2406374 | T | C | 0.015 | 0.002 | 0.314 | 5 | 1.07E+08 | 1.89998e-10 |
| rs2535593 | C | T | 0.013 | 0.002 | 0.46 | 17 | 5621920 | 8.30004e-09 |
| rs2553041 | C | G | 0.02 | 0.003 | 0.196 | 2 | 63238346 | 1e-13 |
| rs2612030 | C | T | 0.024 | 0.003 | 0.838 | 3 | 53773437 | 1.29987e-16 |
| rs2613765 | A | G | 0.013 | 0.002 | 0.473 | 19 | 5066330 | 6.49995e-09 |
| rs2744450 | G | A | 0.017 | 0.003 | 0.802 | 6 | 52951766 | 2.80001e-10 |
| rs293736 | C | A | -0.014 | 0.002 | 0.722 | 20 | 31925189 | 5.69994e-09 |
| rs2974311 | A | G | 0.014 | 0.002 | 0.497 | 8 | 42455166 | 1.40001e-10 |
| rs298247 | C | G | 0.016 | 0.003 | 0.167 | 2 | 1.57E+08 | 1.5e-08 |
| rs3007104 | A | G | -0.015 | 0.002 | 0.424 | 14 | 47367434 | 1.39991e-12 |
| rs30266 | A | G | -0.013 | 0.002 | 0.328 | 5 | 1.04E+08 | 3.69999e-08 |
| rs341521 | A | G | -0.015 | 0.002 | 0.703 | 13 | 60399045 | 1.09999e-10 |
| rs34517439 | A | C | -0.02 | 0.003 | 0.122 | 1 | 78450517 | 2.90001e-09 |
| rs34811474 | A | G | 0.015 | 0.003 | 0.231 | 4 | 25408838 | 2.30001e-09 |
| rs35128508 | A | G | 0.015 | 0.002 | 0.273 | 18 | 42722340 | 1.29999e-09 |
| rs35408390 | T | C | -0.012 | 0.002 | 0.443 | 2 | 86471782 | 2.19999e-08 |
| rs35851551 | G | A | -0.026 | 0.004 | 0.101 | 7 | 31330785 | 2.09991e-13 |
| rs359239 | T | C | -0.019 | 0.002 | 0.574 | 2 | 60474600 | 4.79954e-18 |
| rs369230 | T | G | -0.018 | 0.002 | 0.692 | 16 | 89645437 | 3.90032e-15 |
| rs3739121 | C | G | -0.012 | 0.002 | 0.427 | 2 | 2.01E+08 | 1.7e-08 |
| rs3741499 | C | T | -0.016 | 0.002 | 0.649 | 12 | 56474379 | 8.69961e-13 |
| rs3758790 | G | A | 0.015 | 0.002 | 0.359 | 11 | 1.06E+08 | 8.9002e-12 |
| rs3789045 | T | C | 0.015 | 0.003 | 0.209 | 1 | 2.05E+08 | 1.5e-08 |
| rs3896224 | G | A | 0.021 | 0.002 | 0.415 | 10 | 1.06E+08 | 3.69999e-22 |
| rs4075359 | C | T | 0.019 | 0.002 | 0.625 | 8 | 9487813 | 2.90001e-17 |
| rs435538 | G | C | -0.018 | 0.003 | 0.23 | 5 | 24921617 | 3.40017e-12 |
| rs4443996 | C | A | -0.017 | 0.002 | 0.476 | 10 | 1.34E+08 | 1.10002e-14 |
| rs4602427 | G | C | -0.016 | 0.003 | 0.804 | 3 | 1.17E+08 | 2.5e-09 |
| rs4702 | A | G | 0.017 | 0.002 | 0.556 | 15 | 91426560 | 1.29987e-14 |
| rs4727799 | T | C | -0.021 | 0.002 | 0.647 | 7 | 1.14E+08 | 2.80027e-20 |
| rs4735438 | T | C | -0.014 | 0.002 | 0.587 | 8 | 97829917 | 3.90032e-11 |
| rs4755749 | G | A | 0.016 | 0.002 | 0.403 | 11 | 43865501 | 7.29962e-13 |
| rs4800204 | T | C | -0.013 | 0.002 | 0.57 | 18 | 22647270 | 4.60002e-09 |
| rs4805761 | G | A | 0.017 | 0.003 | 0.843 | 19 | 32951800 | 2.59998e-08 |
| rs4873133 | T | C | -0.018 | 0.002 | 0.292 | 8 | 51127054 | 2.49977e-14 |
| rs4937872 | G | A | -0.016 | 0.002 | 0.408 | 11 | 1.13E+08 | 3.90032e-13 |
| rs4952343 | G | A | 0.014 | 0.002 | 0.446 | 2 | 32858637 | 2.99999e-10 |
| rs4961705 | C | G | 0.013 | 0.002 | 0.348 | 9 | 16347927 | 5e-09 |
| rs55659265 | A | G | -0.024 | 0.004 | 0.067 | 2 | 1.42E+08 | 1.5e-08 |
| rs56306056 | A | G | 0.016 | 0.003 | 0.215 | 2 | 1.84E+08 | 8.60003e-10 |
| rs56392241 | C | A | -0.014 | 0.002 | 0.393 | 3 | 1.32E+08 | 8.60003e-10 |
| rs56393977 | T | G | 0.022 | 0.004 | 0.102 | 18 | 39265197 | 3.59998e-10 |
| rs57537843 | A | G | -0.016 | 0.002 | 0.381 | 2 | 22558973 | 6.00067e-13 |
| rs6011138 | C | T | 0.019 | 0.003 | 0.136 | 20 | 62440700 | 2.30001e-09 |
| rs61746970 | A | G | -0.032 | 0.006 | 0.039 | 19 | 51132746 | 2.39999e-08 |
| rs61864459 | T | G | 0.016 | 0.003 | 0.17 | 10 | 1.2E+08 | 2.1e-08 |
| rs62134195 | T | C | 0.034 | 0.006 | 0.041 | 2 | 45062249 | 6.90001e-10 |
| rs62370848 | C | A | 0.017 | 0.003 | 0.199 | 5 | 1.24E+08 | 3.59998e-10 |
| rs62439690 | A | G | -0.016 | 0.002 | 0.262 | 7 | 21417556 | 6.29941e-11 |
| rs6486065 | G | T | 0.016 | 0.002 | 0.709 | 11 | 12870969 | 1e-11 |
| rs6517512 | G | A | -0.03 | 0.005 | 0.956 | 21 | 40512129 | 1.79999e-08 |
| rs6549670 | G | A | -0.018 | 0.003 | 0.839 | 3 | 74936132 | 4.70002e-10 |
| rs6564268 | G | C | 0.028 | 0.005 | 0.056 | 16 | 75606878 | 1.5e-09 |
| rs66906321 | C | T | -0.018 | 0.003 | 0.819 | 2 | 630995 | 2e-10 |
| rs6692613 | T | C | -0.013 | 0.002 | 0.53 | 1 | 7524974 | 4.20001e-09 |
| rs6719762 | C | T | -0.023 | 0.002 | 0.473 | 2 | 60166832 | 3.19963e-27 |
| rs6744794 | G | C | -0.02 | 0.002 | 0.623 | 2 | 44842145 | 4.30031e-20 |
| rs6748341 | G | C | 0.017 | 0.002 | 0.316 | 2 | 2.25E+08 | 5.40008e-13 |
| rs6763967 | A | G | -0.014 | 0.002 | 0.265 | 3 | 60884616 | 7.90005e-09 |
| rs67723420 | A | T | 0.015 | 0.002 | 0.376 | 3 | 35775115 | 7.50067e-11 |
| rs6939048 | A | G | 0.014 | 0.002 | 0.627 | 6 | 26327953 | 1.29999e-10 |
| rs6955073 | A | T | 0.012 | 0.002 | 0.508 | 7 | 1.18E+08 | 9.80009e-09 |
| rs6966769 | G | A | 0.019 | 0.003 | 0.114 | 7 | 1299334 | 4.90004e-08 |
| rs6966898 | T | C | -0.014 | 0.002 | 0.333 | 7 | 1.35E+08 | 1.6e-09 |
| rs6973256 | T | C | 0.014 | 0.002 | 0.598 | 7 | 1.33E+08 | 2.1e-10 |
| rs6978112 | T | C | -0.017 | 0.002 | 0.412 | 7 | 1966841 | 7.39946e-15 |
| rs702 | T | A | 0.021 | 0.003 | 0.839 | 4 | 28710551 | 2.60016e-12 |
| rs7025089 | A | C | 0.016 | 0.002 | 0.681 | 9 | 1.35E+08 | 3.59998e-12 |
| rs705240 | T | C | -0.016 | 0.003 | 0.184 | 3 | 1.18E+08 | 7.00003e-09 |
| rs7085104 | G | A | -0.015 | 0.002 | 0.329 | 10 | 1.05E+08 | 2e-10 |
| rs710289 | G | A | -0.016 | 0.002 | 0.416 | 14 | 98535256 | 3.19963e-13 |
| rs71433405 | T | C | 0.026 | 0.004 | 0.066 | 13 | 97138668 | 2.99999e-09 |
| rs714393 | T | C | 0.014 | 0.002 | 0.457 | 2 | 2.13E+08 | 6.09958e-11 |
| rs7156339 | T | C | -0.024 | 0.003 | 0.164 | 14 | 1.03E+08 | 4.90004e-16 |
| rs7167444 | T | G | -0.014 | 0.003 | 0.253 | 15 | 97495941 | 1.7e-08 |
| rs7188873 | G | A | -0.017 | 0.002 | 0.623 | 16 | 24727064 | 8.60003e-15 |
| rs7236339 | A | G | -0.02 | 0.003 | 0.228 | 18 | 77579773 | 2.49977e-15 |
| rs72674824 | C | T | 0.015 | 0.003 | 0.242 | 8 | 95489281 | 5.60003e-09 |
| rs72822625 | A | G | 0.022 | 0.004 | 0.095 | 5 | 1.68E+08 | 5.49997e-09 |
| rs72887338 | C | T | -0.016 | 0.002 | 0.387 | 6 | 67536056 | 1.29987e-13 |
| rs72996415 | C | T | 0.025 | 0.004 | 0.065 | 6 | 1.05E+08 | 1.5e-08 |
| rs7381195 | A | T | -0.015 | 0.002 | 0.612 | 5 | 60030791 | 3.80014e-11 |
| rs74583305 | T | C | 0.044 | 0.008 | 0.02 | 8 | 1.43E+08 | 1.09999e-08 |
| rs7476 | C | A | -0.014 | 0.002 | 0.312 | 11 | 46342834 | 2.59998e-09 |
| rs75082770 | G | A | -0.035 | 0.006 | 0.038 | 2 | 1.86E+08 | 6.29999e-10 |
| rs75783371 | C | T | 0.019 | 0.003 | 0.139 | 13 | 28109961 | 3.2e-09 |
| rs767943 | A | C | -0.019 | 0.002 | 0.265 | 6 | 23446691 | 3.59998e-14 |
| rs77214504 | T | A | 0.035 | 0.005 | 0.047 | 1 | 75316394 | 7.10068e-12 |
| rs7729019 | T | C | 0.013 | 0.002 | 0.614 | 5 | 1.56E+08 | 1.09999e-08 |
| rs7785195 | A | G | 0.016 | 0.002 | 0.659 | 7 | 3424686 | 2.99985e-12 |
| rs7804551 | G | A | 0.021 | 0.003 | 0.154 | 7 | 99119110 | 3.29989e-12 |
| rs7815125 | A | T | 0.019 | 0.003 | 0.827 | 8 | 87680112 | 4.30031e-11 |
| rs783544 | C | A | 0.016 | 0.002 | 0.751 | 15 | 83240293 | 4.00037e-11 |
| rs784255 | T | G | -0.014 | 0.002 | 0.475 | 18 | 53403228 | 4.40048e-11 |
| rs7857266 | T | C | 0.013 | 0.002 | 0.395 | 9 | 96381765 | 3.69999e-09 |
| rs7868984 | C | T | 0.014 | 0.002 | 0.416 | 9 | 23357826 | 2.69998e-10 |
| rs7909331 | G | A | -0.017 | 0.003 | 0.165 | 10 | 11205224 | 9.20005e-09 |
| rs7911962 | T | C | 0.014 | 0.002 | 0.384 | 10 | 9972046 | 1.09999e-09 |
| rs79231171 | A | C | 0.022 | 0.004 | 0.077 | 2 | 2.14E+08 | 2.69998e-08 |
| rs79269403 | A | G | 0.02 | 0.003 | 0.231 | 3 | 1.08E+08 | 3.50026e-15 |
| rs794375 | C | T | 0.016 | 0.002 | 0.426 | 7 | 75147801 | 1.29987e-12 |
| rs8003519 | G | A | -0.015 | 0.002 | 0.581 | 14 | 93830357 | 9.3994e-12 |
| rs800532 | G | A | -0.016 | 0.003 | 0.776 | 8 | 1.17E+08 | 1.5e-09 |
| rs803679 | A | G | 0.02 | 0.003 | 0.793 | 1 | 44349405 | 1.10002e-13 |
| rs807478 | G | A | 0.014 | 0.002 | 0.497 | 19 | 36252494 | 4.60045e-11 |
| rs8096225 | C | A | -0.013 | 0.002 | 0.695 | 18 | 36921851 | 1.79999e-08 |
| rs8133065 | G | C | 0.014 | 0.002 | 0.283 | 21 | 31422507 | 8.60003e-09 |
| rs838039 | A | G | 0.015 | 0.002 | 0.687 | 2 | 1.4E+08 | 2.1e-10 |
| rs9491228 | C | T | -0.014 | 0.002 | 0.529 | 6 | 1.25E+08 | 6.70039e-11 |
| rs9514600 | G | C | -0.012 | 0.002 | 0.502 | 13 | 1.08E+08 | 3.40001e-08 |
| rs9536961 | G | A | 0.013 | 0.002 | 0.348 | 13 | 55678332 | 1.2e-08 |
| rs9538248 | A | C | -0.016 | 0.002 | 0.321 | 13 | 59492828 | 1.29987e-11 |
| rs976179 | T | A | -0.012 | 0.002 | 0.485 | 2 | 1.99E+08 | 2.99999e-08 |
| rs9809849 | A | G | -0.014 | 0.002 | 0.425 | 3 | 3726156 | 8.69961e-11 |
| rs9866968 | A | G | 0.024 | 0.002 | 0.349 | 3 | 85683470 | 6.29941e-27 |
| rs9873182 | G | C | -0.026 | 0.003 | 0.841 | 3 | 88250597 | 2.29985e-18 |
| rs9886840 | G | A | 0.013 | 0.002 | 0.58 | 9 | 1.25E+08 | 1e-08 |
| rs9891146 | C | T | 0.014 | 0.002 | 0.735 | 17 | 65988049 | 6.29999e-09 |
| rs9904818 | C | G | 0.022 | 0.004 | 0.09 | 17 | 77793292 | 3.29997e-09 |
| rs9923553 | G | A | -0.014 | 0.002 | 0.29 | 16 | 5825579 | 1.7e-09 |
| rs9964201 | A | C | 0.026 | 0.004 | 0.083 | 18 | 50600552 | 1.50003e-11 |
